# The association between experiencing homelessness in childhood or youth and adult housing stability in Housing First

**DOI:** 10.1186/s12888-021-03142-0

**Published:** 2021-03-08

**Authors:** Milad Parpouchi, Akm Moniruzzaman, Julian M. Somers

**Affiliations:** Somers Research Group, Faculty of Health Sciences, Simon Fraser University, Blusson Hall, Room 11300, 8888 University Drive, Burnaby, British Columbia V5A 1S6 Canada

**Keywords:** Homelessness, Mental illness, Housing stability, Housing First, Canada

## Abstract

**Background:**

Researchers have pointed out the paucity of research investigating long-term consequences of experiencing homelessness in childhood or youth. Limited research has indicated that the experience of homelessness in childhood or youth is associated with adverse adjustment-related consequences in adulthood. Housing First (HF) has acknowledged effectiveness in improving housing outcomes among adults experiencing homelessness and living with serious mental illness, although some HF clients struggle with maintaining housing. The current study was conducted to examine whether the experience of homelessness in childhood or youth increases the odds of poorer housing stability following entry into high-fidelity HF among adults experiencing serious mental illness and who were formerly homeless.

**Methods:**

Data were drawn from the active intervention arms of a HF randomized controlled trial in Metro Vancouver, Canada. Participants (*n* = 297) were referred to the study from service agencies serving adults experiencing homelessness and mental illness between October 2009 and June 2011. The Residential Time-Line Follow-Back Inventory was used to measure housing stability. Least absolute shrinkage and selection operator was used to estimate the association between first experiencing homelessness in childhood or youth and later housing stability as an adult in HF.

**Results:**

Analyses indicated that homelessness in childhood or youth was negatively associated with experiencing housing stability as an adult in HF (aOR = 0.53; 95% CI = 0.31–0.90).

**Conclusions:**

Further supports are needed within HF to increase housing stability among adult clients who have experienced homelessness in childhood or youth. Asking clients about the age they first experienced homelessness may be of clinical utility upon enrollment in HF and may help identify support needs related to developmental experiences. Results further emphasize the importance of intervening earlier in life in childhood and youth before experiencing homelessness or before it becomes chronic. Findings also contribute to a limited knowledge base regarding the adverse long-term consequences of childhood and youth homelessness.

**Trial registration:**

Current Controlled Trials: ISRCTN57595077 and ISRCTN66721740. Registered on October 9, 2012.

**Supplementary Information:**

The online version contains supplementary material available at 10.1186/s12888-021-03142-0.

## Background

Homelessness has become a public health crisis in North America. In Canada, it has been estimated that 235,000 people experience homelessness each year, with 35,000 people experiencing it each night. About 20% are unaccompanied youth ages 13–24, equating to approximately 35,000–40,000 people [1]. A national point-in-time homeless count in the U.S. found about 553,000 people experiencing homelessness in 2018, and nearly 7 % were unaccompanied youth under the age of 25, equating to approximately 36,000 people [2]. Moreover, over 1.35 million children enrolled in elementary and secondary public schools in the 2016–2017 school year were reported as homeless in the U.S. alone [3]. About 2.5 million children under the age of 18 are estimated to be experiencing homelessness on an annual basis in the U.S. [4].

Homelessness has increased since the 1980s in many Western countries, and demographic changes have accompanied this increase within the population [1, 5, 6]. As one example, in Canada, a greater proportion of people experiencing homelessness are youth than was the case prior to the 1980s [1]. Furthermore, families and children among people experiencing homelessness are increasing at the highest rate [7]. In the U.S., families with children now comprise one-third of people experiencing homelessness [2], and this figure has also grown since the 1980s [6].

Youth experiencing homelessness may develop skills necessary to live on the streets [8], but may not have yet had opportunities to develop the life skills necessary for living independently [8, 9]. Additionally, researchers have hypothesized that experiencing homelessness earlier in life, during sensitive developmental years, may have more harmful consequences later in life [10]. Kilmer and colleagues have argued that the stressors involved in the experience of homelessness during childhood may increase “the likelihood that youngsters will evidence difficulties as they move along their adjustment trajectories” [11] p. 391. Similar arguments have been made regarding development for those experiencing homelessness in youth [12].

Numerous studies have found that homelessness during childhood or youth is associated with a myriad of health and social problems, including, infectious disease, chronic physical health conditions, poor nutrition, dental disease, mental illness, substance abuse, injury, mortality, poorer cognitive functioning and academic performance, behavioral health risks, and violence [12–14]. A systematic review of studies using “full psychiatric diagnostic interview [s]” found the prevalence of psychiatric disorders among youth experiencing homelessness to be between 48 and 98% ([15], p. e3). Moreover, the experience of homelessness as a very young child may also be associated with adverse consequences, such as developmental delays. For example, one study found that infants and children aged 2 months to 6 years experiencing homelessness had developmental scores at levels significantly poorer than the general population, with the most pronounced differences in the domains of language and communication [16].

Findings from some longitudinal studies suggest that experiencing homelessness earlier in life may be independently and significantly associated with adverse consequences later in life. For example, one prospective study among participants aged 18–26 found that the experience of homelessness before the age of 26 was independently and significantly associated with an increased likelihood of committing a violent or property crime later in adulthood [17]. Similarly, using panel data among a representative sample of people in Australia at least 15 years of age who had experienced homelessness or were at risk, Cobb-Clark and Zhu [10] found that the experience of homelessness first in childhood (≤15 years of age) among men aged 21–54 years in the study was significantly associated with a decreased likelihood of employment in adulthood in their adjusted model, compared to the men who had first experienced homelessness later in their life. When potential mediating variables were considered, there was still a direct effect. Using eight-year follow-up data from the National Longitudinal Survey of Youth-Child Study, Stablein and Appleton [18] compared the health of adolescents and young adults who were formerly homeless (ages 15–25) to those who had not experienced homelessness. They found that the experience of homelessness (occurring any time between 2000 and 2006) was significantly associated with having an incident case of asthma, a health-limiting condition, and developing poorer self-rated health following homelessness at the final follow-up (2008). The relationship between homelessness and asthma and having a health-limiting condition was partly mediated by other variables, but the relationship with self-rated health remained independent. The experience of homelessness was also independently and significantly associated with lower education attainment, an increased risk of depressive symptoms, and alcohol and substance abuse following homelessness. A cross-sectional study conducted in Canada similarly found that the earlier the age of first experiencing homelessness, the higher the likelihood of being in high psychological distress among a large sample of youth accessing services for people experiencing homelessness [19].

In light of the above longitudinal studies reporting adverse long-term consequences of homelessness among children and youth, it may be the case that once an adult, people may have difficulty adjusting to housing and support interventions. One such intervention may be Housing First (HF).

HF is a supported housing model that brings together permanent housing and health and social services for people experiencing homelessness and serious mental illness [20, 21]. Based on the psychosocial rehabilitation model (also called psychiatric rehabilitation) [22–24], clients of HF are provided choice and can decide what, if any, services to engage in while in the program (e.g., mental health treatment) [25]. Such choice is also extended to substance use, as there are no programmatic requirements regarding abstinence [20].

Since its establishment in New York City [20], HF has been implemented in many parts of the globe, including, but not limited to, Canada [26], Australia [27], and Europe [28]. Systematic reviews have consistently found HF to be associated with increased housing stability outcomes [29–32]. In a recent review of tenancy sustainment following homelessness, Boland et al. [31], concluded “that Housing First is the most promising intervention” [p. e6].

What is less often discussed in the literature is the proportion of people for whom HF does not help to maintain housing stability. Volk et al. [33] noticed a trend in the literature; about 15–20% of participants in HF studies exhibit housing instability. A few studies have quantitatively investigated factors associated with housing instability, retention, or relocations within HF [33–38], however, to our knowledge, no studies have reported the association between first experiencing homelessness in childhood or youth and subsequent housing stability in adulthood within HF. Adair et al. [38] included the age of first experiencing homelessness as a predictor in their modelling approach, but results were not reported for this specific variable. More broadly, other researchers have pointed out the paucity of research investigating long-term consequences of experiencing homelessness in childhood or youth [10].

The objective of the current study was to examine the association between having first experienced homelessness in childhood or youth and housing stability following the implementation of high-fidelity HF among adults experiencing homelessness or precarious housing and living with serious mental illness. We hypothesized that the experience of homelessness in childhood or youth would be significantly and independently associated with poorer housing stability after receiving HF as an adult. Such research is important to: 1) contribute to the understanding of risk for poorer housing stability after receiving HF 2) improve policies and practices related to the intervention, and 3) add to the limited literature regarding long-term consequences of childhood and youth homelessness.

## Methods

### Participants and sampling

The present study is based on a larger experimental investigation called Vancouver At Home (VAH), which included two pragmatic randomized controlled field trials involving two years of follow-up (Current Controlled Trials: ISRCTN57595077 and ISRCTN66721740). The trials examined HF in congregate and scattered-site configurations among adults experiencing homelessness or precarious housing and living with serious mental illness (*n* = 497) in Metro Vancouver, British Columbia, Canada. Interventions were compared to treatment as usual (TAU). The protocol for VAH has been published [39].

Participants were referred to the study from service agencies serving adults experiencing homelessness and mental illness (e.g., homeless shelters) in Metro Vancouver, Canada between October 2009 and June 2011. Study eligibility included: being a Canadian citizen, at least 19 years of age, absolutely homeless or precariously housed, and having a serious mental illness (assessed by the Mini International Neuropsychiatric Interview [MINI]) [40]. VAH considered participants with “no fixed place to sleep or live for more than 7 nights and little likelihood of obtaining accommodation in the coming month” as absolutely homeless [39] p. 3. Participants “currently residing in marginal accommodation, such as a SRO [single-room occupancy] hotel, and having two or more episodes of [absolute] homelessness (as defined above) during the past 12 months” were considered precariously housed [39] p. 3. Written informed consent was provided by participants.

Once enrolled in the study, a range of interviewer-administered questionnaires were used to elicit information from participants at baseline, including, but not limited to, socio-demographics, service use, mental disorders and symptoms, community functioning, physical comorbidities, and substance use. Data collected during the baseline interview were also used to determine participant support need levels. A comprehensive assessment algorithm was used to differentiate participants with “high needs” from “moderate needs”. Criteria determining participants with high needs included the presence of a psychotic disorder or bipolar disorder (according to the MINI) [40], receiving a score of ≤62 on the Multnomah Community Ability Scale [41], and one of the following: having a history of arrest or incarceration in the past six months (using the Demographics, Service & Housing History [DSHH] questionnaire; see Additional file 1), two or more psychiatric hospitalizations in one of the past 5 years (using the Screener questionnaire; see Additional file 2), or substance dependence (according to the MINI) [40]. All other participants were considered as having moderate needs. Additional information about VAH, such as sampling and questionnaires used, have been published [see 39]. The study underwent ethics review and was approved by the Research Ethics Board of Simon Fraser University. The study was also conducted as per relevant guidelines and regulations.

### Interventions

The two VAH randomized controlled trials were differentiated based on participant need levels (i.e., moderate vs. high needs). The randomized controlled trial for those with moderate needs randomly allocated participants to either scattered-site HF with intensive case management (HF-ICM) or TAU (comprised of existing services in the community). The randomized controlled trial for those with high needs randomly allocated participants to scattered-site HF with assertive community treatment (HF-ACT), congregate HF with on-site support (CONG), or TAU. A description of each HF intervention follows.

The HF-ICM intervention included a private rental apartment of the participant’s choice in Metro Vancouver combined with intensive case management whereby case managers helped participants access existing community services and were available 12 h a day. HF-ACT included the same housing as HF-ICM, but the support service component included a multi-disciplinary health and social service provider team located in the community and available 24/7. The CONG intervention involved a single building all occupied by study participants. Participants were provided an independent room and bathroom, but other spaces were shared with tenants (e.g., kitchen). The support component of CONG involved on-site health and social services available 24/7. Moreover, a range of recreational and volunteer activities were provided as part of the intervention. Somers et al. [39] have published additional information on interventions and randomization procedures. We only included participants randomized to intervention arms (i.e., HF-ICM, HF-ACT, or CONG) in the current study.

### Variables of interest

The main outcome was housing stability and was measured using the Residential Time-Line Follow-Back Inventory every three months [42]. Validity of the Residential Time-Line Follow-Back Inventory has been demonstrated among people experiencing homelessness and serious mental illness, with administrative data from agencies providing housing and support used as the reference comparison [42]. Stable housing was defined as having tenancy rights or living in one’s own apartment/room/house/family for an expected time of at least 6 months. Unstable housing was defined as living on the streets or in temporary accommodations, including, but not limited to shelters, hospitals, and crisis units [43]. For the present study, we operationalized housing stability as participants spending ≥90% of days in stable housing during the 2-year follow-up period. Participants spending < 90% of days in stable housing were considered unstably housed. Researchers have argued that definitions of housing stability in the literature are widely inconsistent e.g., [33, 42]. The ideal purpose of HF is to eliminate homelessness and facilitate stable housing, however, as Pearson et al. [34] argue, “housing stability in Housing First programs is an iterative process [and] temporary departures from housing are not uncommon … These episodic departures are part of a stabilizing strategy to ensure that clients maintain their engagement in housing and treatment” [p. 415]. Given that HF clients are among the most vulnerable and marginalized of people experiencing homelessness and that temporary exits from the program may be part of the journey to recovery, we decided on 90% as the stable housing cut-off.

The primary independent variable was age of first experiencing homelessness and was asked during the study’s baseline interview using the DSHH (see Additional file 1 for this questionnaire). Youth have been commonly defined as up to the age of 24 or 25 e.g., [13, 44–46]. The United Nations [45] defines youth as 15–24 years of age and children as below this age (< 14 years of age). Because we were interested in the experience of homelessness in childhood or youth, we operationalized our primary independent variable as age of first experiencing homelessness and dichotomized it as < 25 years vs. ≥25 years.

Intervention type and other relevant variables at baseline were included per prior literature as control covariates. Apart from the type of HF intervention variable (HF-ICM, HF-ACT, CONG), socio-demographic variables were asked from the DSHH and included gender (woman, man), ethnicity (Indigenous, White, Other), education (less than high school, high school or higher), and marital status (single and never married, other). Age at randomization (< 25 years, 25–44 years, > 44 years) was asked from the Screener (see Additional file 2 for this questionnaire). Lifetime duration of homelessness (≤ 36 months, > 36 months) and longest episode of homelessness (≤12 months, > 12 months) were asked from the DSHH, while housing status at enrollment (absolutely homeless, precariously housed) was asked from the Screener. Criminal justice variables were asked from the DSHH and included the following two, occurring anytime in the 6 months preceding baseline: 1) having been arrested (> 1), imprisoned (≥1), on probation, or received a community sanction (yes/no) and 2) having spent at least one night in jail (yes/no). Mental illness variables included mental health symptom severity (Colorado Symptom Index score; higher scores indicate greater symptom severity) [47], the less severe cluster of mental disorders (yes/no; includes at least one of: major depressive episode, panic disorder, or post-traumatic stress disorder according to the MINI) [40], and the severe cluster of mental disorders (yes/no; includes at least one of: psychotic disorder, mood disorder with psychotic features, or manic or hypomanic episode according to the MINI) [40]. Learning disability variables included perceiving having had a learning disability in childhood (yes/no) and having been told of having a learning disability in childhood (yes/no) and were asked from the Screener. Community functioning was determined by the Multnomah Community Ability Scale score (interviewer-rated; higher scores indicate greater community functioning) [41]. Substance use behaviours and income related to sex work in the month preceding baseline were asked from the Maudsley Addiction Profile [48] and included use of alcohol (yes/no), heroin (yes/no), illicit methadone (yes/no), benzodiazepines (yes/no), cocaine (yes/no), crack (yes/no), amphetamine (yes/no), cannabis (yes/no), injection of drugs (yes/no), daily substance use (yes/no; including alcohol), daily drug use (yes/no; excluding alcohol), daily hard drug use (yes/no; excluding alcohol and cannabis), and sex work-related income (yes/no). Money spent on alcohol (yes/no; in Canadian dollars) and money spent on drugs (yes/no; in Canadian dollars) were asked from the Global Assessment of Individual Need (Substance Problem Scale) [49].

Three additional control covariates were included but were not collected at study baseline. Experiences of family separation and foster care were asked at the 12-month follow-up interview from the Vancouver Foster Care questionnaire used in a previous VAH study [50]. The two variables included having lived away from parents for any reason under the age of 18 (yes/no) and having ever been in foster care (yes/no). Information related to adverse childhood experiences was asked from the Adverse Childhood Experiences questionnaire [51] at the 18-month follow-up interview (calculated as a total score up to 10 corresponding to the number of categories of adverse experiences reported). Information related to all questionnaires administered in VAH have been published [39]. All VAH questionnaires were interviewer-administered in person.

### Statistical analysis

Means and standard deviations were presented for continuous variables, and percentages were presented for categorical variables. Least absolute shrinkage and selection operator (LASSO) was used to model the primary independent variable of interest (i.e., age of first experiencing homelessness) and housing stability. LASSO is a regression analysis method which is used in selecting and fitting variables for a statistical model with a large set of potential covariates [52]. It uses a modern data driven method that selects only a subset of the provided variables for the model and tests them in other datasets in order to improve the prediction accuracy and interpretability of regression models. Moreover, LASSO can be used to make inference about the variable of interest in the presence of many potential control covariates [53, 54]. Among LASSO, the cross-fit partialing-out method was specified. This method, which is also known as Double Machine Learning, has a better finite sample property and is more robust, due to the cross-fit nature (coefficients are obtained from one sample and used in another, and this procedure is repeated several times) and split sample technique [54, 55]. In the current analysis, the effect (odds ratio) of the age in which participants first experienced homelessness on housing stability was estimated using binary logistic regression. In the LASSO model, age of first experiencing homelessness was used as the primary independent variable of interest and all other variables (e.g., age, gender, ethnicity, etc.) and their interaction terms were used as controlling covariates. The two variables related to family separation (i.e., having lived away from parents for any reason under the age of 18 and having been in foster care) and the variable pertaining to adverse childhood experiences were not included as control covariates in the primary LASSO multivariable model because data for these variables were collected at later follow-up interviews than the other independent variables, which were collected at baseline. It was also expected that the inclusion of these variables would result in a substantially reduced sample size since they were asked later on in the study. However, in order to control for these variables, two separate LASSO models were conducted as sensitivity analyses, one of which included the two family separation-related variables in addition to the other control covariates originally included in the primary multivariable model. The second sensitivity analysis included the adverse childhood experiences variable in addition to the other control covariates originally included in the primary multivariable model. *P*-values less than 0.05 were considered significant. Missing values were low (< 1.5%) – with the exception of the use of cannabis variable (this variable was added after study recruitment had begun; 9.4% missing) – and were replaced by median values for continuous variables and by largest group for categorical variables. For the outcome, housing stability, the last observation was carried forward in the event of missing data. Stata 16 [56] was used to conduct these analyses.

The follow-up period included the date of randomization until the last available follow-up interview. Follow-up rates are shown in Somers et al. [39].

## Results

**Fig. 1:**
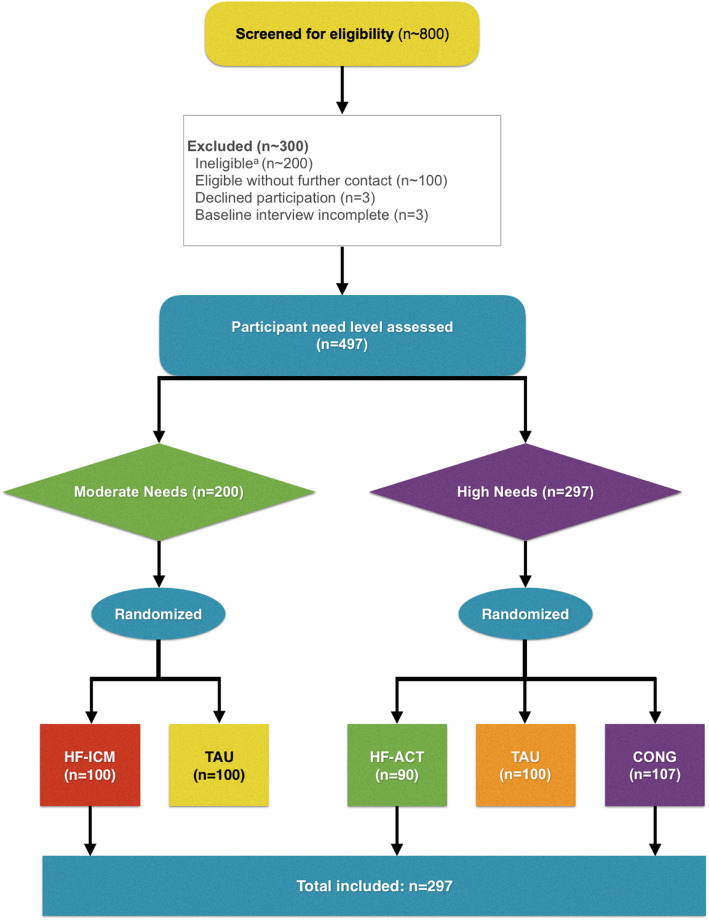
**Flow of participants.** ^a^Includes about 100 participants ineligible after telephone screening, and 94 participants after in-person screening. Abbreviations: HF-ICM = Housing First with intensive case management; TAU = Treatment as usual; HF-ACT = Housing First with assertive community treatment; CONG = Congregate Housing First with on-site support.

Overall, 497 participants were enrolled into VAH. Of these participants, 297 (60%) were randomized to HF intervention arms, including 100 to HF-ICM, 90 to HF-ACT, and 107 to CONG. Figure 1 presents the flow of participants. About 44% of participants reported first experiencing homelessness before the age of 25. Results for additional characteristics of participants are listed in Table 1.

**Table 1:** Characteristics of Vancouver At Home participants randomized to Housing First (*n* = 297)

Variable	***n*** (%) / mean (SD)
***Socio-demographics***	
Age at randomization	
< 25 years	22 (7.4)
25–44 years	171 (57.6)
> 44 years	104 (35.0)
Gender	
Woman	77 (26.0)
Man	219 (74.0)
Ethnicity	
Indigenous	51 (17.2)
White	173 (58.2)
Other	73 (24.6)
Education	
Less than high school	173 (58.8)
High school or higher	121 (41.2)
Marital Status	
Single (never married)	205 (69.5)
Other	90 (30.5)
***Homelessness***	
Age of first homeless	
< 25 years	128 (43.7)
≥ 25 years	165 (56.3)
Lifetime duration of homelessness	
≤ 36 months	145 (49.5)
> 36 months	148 (50.5)
Longest episode of homelessness	
≤ 12 months	141 (48.1)
> 12 months	152 (51.9)
Housing status (at enrollment)	
Absolutely homeless	232 (78.1)
Precariously housed	65 (21.9)
***Criminal justice (past 6 months)***	
Arrested (> 1), imprisoned (≥1), on probation, or received community sanction	
No	161 (54.2)
Yes	136 (45.8)
Spent at least one night in jail	
No	256 (86.2)
Yes	41 (13.8)
***Mental illness, learning disability, and community functioning***	
Mental health symptom severity (Colorado Symptom Index score)	
Mean (SD)	36.6 (12.8)
Less severe cluster of mental disorders	
No	147 (49.5)
Yes	150 (50.5)
Severe cluster of mental disorders	
No	75 (25.2)
Yes	222 (74.8)
Learning disability in childhood (you perceived)	
No	190 (64.0)
Yes	107 (36.0)
Learning disability in childhood (someone told you)	
No	191 (64.3)
Yes	106 (35.7)
Community functioning (Multnomah Community Ability Scale score)	
Mean (SD)	55.2 (9.5)
***Family separation and adverse childhood experiences***	
Lived away from parents for any reason under the age of 18^1^	
No	110 (40.1)
Yes	164 (59.9)
Ever placed in foster care^2^	
No	190 (69.3)
Yes	84 (30.7)
Adverse childhood experiences (ACE)^3^	
Mean (SD)	3.8 (2.7)
***Substance use (past month)***	
Use of alcohol	
No	164 (55.6)
Yes	131 (44.4)
Use of heroin	
No	240 (81.4)
Yes	55 (18.6)
Use of illicit methadone	
No	283 (95.9)
Yes	12 (4.1)
Use of benzodiazepines	
No	268 (91.2)
Yes	26 (8.8)
Use of cocaine	
No	245 (83.1)
Yes	50 (16.9)
Use of crack	
No	197 (66.8)
Yes	98 (33.2)
Use of amphetamine	
No	254 (86.7)
Yes	39 (13.3)
Use of cannabis	
No	148 (55.0)
Yes	121 (45.0)
Injected drugs	
No	239 (81.6)
Yes	54 (18.4)
Daily substance use (including alcohol)	
No	211 (71.5)
Yes	84 (28.5)
Daily drug use (excluding alcohol)	
No	218 (73.9)
Yes	77 (26.1)
Daily hard drug use (excluding alcohol & cannabis)	
No	251 (85.1)
Yes	44 (14.9)
Money spent on alcohol (CAD)	
Mean (SD)	60.0 (164.5)
Money spent on drugs (CAD)	
Mean (SD)	331.3 (852.9)
***Other***	
Sex work-related income (past month)	
No	282 (94.9)
Yes	15 (5.1)
Type of Housing First intervention	
Housing First with intensive case management	100 (33.7)
Housing First with assertive community treatment	90 (30.3)
Congregate housing with on-site support	107 (36.0)

^1^ The variable “Lived away from parents for any reason under the age of 18” was asked at the 12-month follow-up interview, with information available for 274 participants

^2^ The variable “Ever placed in foster care” was asked at the 12-month follow-up interview, with information available for 274 participants

^3^ The variable “Adverse childhood experiences” was asked at the 18-month follow-up interview, with information available for 229 participants

Housing stability outcomes are listed in Table 2. Overall housing stability was 0.73 (SD = 0.27) and was similar by HF intervention, including 0.72 (SD = 0.30) for HF-ICM, 0.74 (SD = 0.25) for HF-ACT, and 0.74 (SD = 0.26) for CONG. About 40% of participants spent ≥90% of days in stable housing during the two years of follow-up, including 44% of participants in HF-ICM, about 36% in HF-ACT, and about 39% in CONG.

**Table 2:** Housing stability among Vancouver At Home participants by Housing First intervention (*n* = 297)

	Overall	HF-ICM (***n*** = 100)	HF-ACT (***n*** = 90)	CONG (***n*** = 107)
Housing stability				
Mean (SD)	0.73 (0.27)	0.72 (0.30)	0.74 (0.25)	0.74 (0.26)
Housing stability, *n* (%)				
< 90% of days	179 (60.3)	56 (56.0)	58 (64.4)	65 (60.8)
≥ 90% of days	118 (39.7)	44 (44.0)	32 (35.6)	42 (39.3)

*Abbreviations*: *HF-ICM* Housing First with intensive case management, *HF-ACT* Housing First with assertive community treatment, *CONG* Congregate Housing First with on-site support

Table 3 presents results from the primary unadjusted and adjusted LASSO analyses. Prior to adjustment with control variables, participants who experienced homelessness < 25 years of age were half as likely to have spent ≥90% of days in stable housing (uOR = 0.50; 95% CI = 0.31–0.81). This result remained statistically significant following adjustment with control variables (aOR = 0.53; 95% CI = 0.31–0.90).

**Table 3:** Least absolute shrinkage and selection operator analyses to estimate the association between first experiencing homelessness in childhood or youth (< 25 years) and housing stability (≥90%) among Vancouver At Home participants randomized to Housing First (*n* = 297)

	Unadjusted Odds Ratio (95% CI)	***P*** value	Adjusted Odds Ratio (95% CI)	P value
Age of first homeless				
< 25 years	**0.50 (0.31, 0.81)**	**0.005**	**0.53 (0.31, 0.90)**	**0.020**
≥ 25 years	Reference		Reference	

Table 4 presents results from both sensitivity analyses. In the first sensitivity analysis, two family separation-related variables (having lived away from parents for any reason under the age of 18 and having ever been in foster care) were added as control covariates to the ones that had been included in the primary multivariable model. The sample size was reduced (*n* = 274), and the experience of homelessness < 25 years of age remained significantly and negatively associated with housing stability both in the unadjusted (OR = 0.51; 95% CI = 0.31–0.85) and adjusted models (aOR = 0.54; 95% CI: 0.31–0.95).

**Table 4:** Sensitivity analyses - least absolute shrinkage and selection operator analyses to estimate the association between first experiencing homelessness in childhood or youth (< 25 years) and housing stability (≥90%) among Vancouver At Home participants randomized to Housing First

Control Covariates Added	Primary Independent Variable	Unadjusted Odds Ratio (95% CI)	***P*** value	Adjusted Odds Ratio (95% CI)	P value
Family separation (*n* = 274)^1^	Age of first homeless				
	< 25 years	**0.51 (0.31, 0.85)**	**0.009**	**0.54 (0.31, 0.95)**	**0.032**
	≥ 25 years	Reference		Reference	
Adverse childhood experiences score (*n* = 229)^2^	Age of first homeless				
	< 25 years	**0.55 (0.32, 0.96)**	**0.035**	**0.60 (0.33, 1.11)**	**0.103**
	≥ 25 years	Reference		Reference	

^1^In addition to control covariates included in the primary LASSO multivariable model, two family separation variables were added: 1) “Lived away from parents for any reason under the age of 18” (yes/no) and 2) “Ever placed in foster care” (yes/no)

^2^In addition to control covariates included in the primary LASSO multivariable model, the following variable was added: “Adverse childhood experiences” (score)

In the second sensitivity analysis, the adverse childhood experiences total score was added as a control covariate to the ones that had been included in the primary multivariable model. This reduced the sample size (*n* = 229) and resulted in a slightly attenuated effect size (OR = 0.60; 95% CI: 0.33–1.11) compared to the primary multivariable model. The *p*-value also became marginally significant (*p* = 0.103).

## Discussion

Consistent with our hypothesis, the experience of homelessness in childhood or youth was significantly and independently associated with lower odds of experiencing housing stability in HF. More specifically, compared to participants who had first experienced homelessness at age 25 or older, participants who had first experienced homelessness under the age of 25 had half the odds of experiencing housing stability as an adult in HF over 24 months.

This finding adds to limited research demonstrating long-term consequences of homelessness for children and youths [10, 17] and underscores the additional support needs of participants in HF. It has been suggested that exposure to and integration into “homeless subculture” [57] p. 578 in childhood or youth via street survival skills, and friendships developed with other people experiencing homelessness and the subsequent “camaraderie” make it more difficult to exit homelessness [57] p. 576. As Johnson and Chamberlain [57] argue, “without a meaningful role to perform and new social networks to engage with, some people find it difficult to disengage from the homeless subculture when it is their primary social network” [p. 578]. These findings are supported by qualitative analyses of VAH outlining lack of meaningful activity and work, boredom, and social isolation following randomization to HF, albeit these data were not broken down by age of first experiencing homelessness [58].

Additionally, studies from the psychosocial rehabilitation and occupational therapy literature dating back as far as the 1980s [8, 59] outlined a range of barriers youth experiencing homelessness faced to independent living, including, but not limited to, unemployment and lack of employment skills, educational deficits, mental health problems, problematic substance use, inadequate social support, and family problems [59]. Helfrich et al. [8] further argue that youth experiencing homelessness “have limited opportunities to develop life skills that promote mainstream roles such as that of student, family member or worker” [p. 191]. These same skill deficits may persist into adulthood without adequate supports. Participants who had experienced homelessness in childhood or youth may have had a more difficult time developing these skills while in VAH HF interventions. Findings of the present study warrant replication and may expose an important area of further research examining the consequences of developmental experiences as they contribute to housing stability.

Research is also needed to determine what modifications and additional support services are needed within HF interventions to increase housing stability for adults who first experienced homelessness in childhood or youth, but two additional implications can also be drawn from the present study: 1) our analyses suggest that gathering information about age of first experiencing homelessness may be of clinical utility upon enrollment in HF, and may help identify support needs related to developmental challenges and experiences, and 2) it is vital to implement housing and support interventions targeting children and youth with or without family members before they become homeless or immediately after becoming so. Other researchers have called for HF as a potential solution among youth experiencing homelessness [60].

A multisite, experimental investigation, which included VAH as one of its sites, previously found HF to be associated with significantly improved housing stability compared to TAU among youth ages 18–24 living with serious mental illness. Results were similar when compared to those older than 24 years of age. However, secondary and exploratory outcomes of the same study were not as promising, with HF even being associated with significantly decreased rates of employment relative to TAU [46]. Differences in needs between youth and adults have led to adaptations of HF specifically for youth [61, 62]. However, there is a paucity of research examining HF among youth experiencing homelessness [62], and existing research on youth experiencing both homelessness and serious mental illness has found that some do not prefer to live independently due to isolation, continued substance use challenges, and potential cultural-related factors, such as leaving one’s existing social circle [9]. Further research is needed to clarify modifications to HF that best support housing stability for youth experiencing homelessness.

There also exists extremely limited research in the area of housing and support interventions for children in families experiencing homelessness. Limited research suggests HF, permanent supportive housing, and housing subsidies or affordable housing are effective in improving housing status [63–65], but reaching housing stability in the long-term has been identified as an unsolved problem [64], with further investigation urgently needed [63–65]. One large multisite study involving 3 y of follow-up comprehensively studied the effects of random assignment to long-term rent subsidies, short-term rent subsidies, and transitional housing combined with support services all compared to TAU among families experiencing homelessness in the U.S. Findings strongly favoured the long-term rent subsidy intervention compared to TAU, with significantly reduced homelessness, increased housing stability, and a variety of improved outcomes among children, including, but not limited to, a significantly reduced percentage of families with ≥1 child separated in the past 6 months at 20 months of follow-up, fewer school absences at 20 months of follow-up, reduced behavioral problems at 37 months of follow-up, and increased food security at 20 and 37 months of follow-up [66].

Beyond HF, other policies and services that address childhood and youth homelessness should be implemented at the earliest possible in order to prevent chronic adverse health and social consequences [67]. As Gaetz et al. [67] outline, these efforts should target primary prevention (e.g., affordable and social housing availability, services to prevent adverse childhood experiences in families, and supports while transitioning out of foster and other institutional care), secondary prevention (e.g., emergency rental funds [68]), and tertiary prevention (e.g. housing and health and social services provided to children, youth, and families experiencing homelessness).

The present study involved several limitations. Although results were similar when the two family separation-related variables were added to the primary multivariable model in the first sensitivity analysis, the same was not the case for the second sensitivity analysis. Specifically, when the adverse childhood experiences variable was added to the primary adjusted model in the second sensitivity analysis, the relationship between first experiencing homelessness in childhood or youth and housing stability became marginally significant, with the same directional relationship as observed in the initial model. However, this was likely due to the considerably reduced sample size (*n* = 229), which also resulted in a wider 95% confidence interval. Additional studies with larger sample sizes are needed to confirm results of the present analyses. It is also important for future research to examine and identify mediators of the relationship between first experiencing homelessness in childhood or youth and poorer housing stability as an adult in Housing First. Another limitation involved insufficient statistical power to include transgender and transsexual as separate variable levels, as one participant self-identified as transgender and another as transsexual. With the exception of the Multnomah Community Ability Scale [41], all variables were self-reported and may have been influenced by social desirability and recall bias. However, previous analyses have demonstrated validity of self-report from participants of VAH [69]. Additionally, follow-up was limited to two years. Longer follow-up is needed to assess the stability of the differences we observed. Lastly, probability sampling was not employed in VAH, limiting generalizability.

## Conclusion

To our knowledge, no studies have investigated the effect of experiencing homelessness in childhood or youth on later housing stability as an adult in HF, and hence, the present study is the first of its kind. We found that participants who had first experienced homelessness in their childhood or youth had about half the odds of experiencing housing stability within HF as defined by spending at least 90% of days in stable housing. This association was both significant and independent. Our findings have implications for service delivery in HF. They also emphasize the importance of intervening earlier in life in childhood and youth before experiencing homelessness and before it becomes chronic, potentially resulting in poorer health and social outcomes. Future research should investigate how best to support adults experiencing housing instability within HF, and how to intervene to best support the housing and related health and social needs of children and youth experiencing homelessness.

## Supplementary Information

**Additional file 1:.** Demographics, Service & Housing History questionnaire; description of data: provides details of the questionnaire and lists its items.

**Additional file 2:.** Screener questionnaire; description of data: provides details of the questionnaire and lists its items.

## Data Availability

The datasets used and/or analyzed during the current study are available from the third author (JMS) on reasonable request at jsomers@sfu.ca.

## Abbreviations

HF: Housing First
VAH: Vancouver At Home
TAU: Treatment as usual
MINI: Mini International Neuropsychiatric Interview
DSHH: Demographics, Service & Housing History
HF-ICM: Housing First with intensive case management
HF-ACT: Housing First with assertive community treatment
CONG: Congregate Housing First with on-site support
LASSO: Least absolute shrinkage and selection operator
