# Supplementary material for: The association between experiencing homelessness in childhood or youth and adult housing stability in Housing First
Source: BMC Psychiatry. 2021 Mar 8;21:138. doi: 10.1186/s12888-021-03142-0 (PMC7938606; doi:10.1186/s12888-021-03142-0)
Supplement: Supplementary file 1 — Additional file 1:. Demographics, Service & Housing History questionnaire; description of data: provides details of the questionnaire and lists its items. [file 12888_2021_3142_MOESM1_ESM.pdf]

The Association Between Experiencing Homelessness in Childhood or Youth and Adult Housing Stability in Housing First

Milad Parpouchi[a], Akm Moniruzzaman[a], Julian M. Somers[a]

[a] Somers Research Group, Faculty of Health Sciences, Simon Fraser University

Demographics, Service & Housing History questionnaire

- Author: At Home/Chez Soi Study National Research Team
  - Author affiliation: Department of Psychiatry, University of Toronto
- Demographic items of this questionnaire were adapted from multiple sources [1-3]
- Please see next page for questionnaire items

References

1. Goering P. Making a difference: Ontario's Community Mental Health Evaluation Initiative. Toronto; 2004.
2. Toronto Board of Education, Organizational Development Department, Research and Information Services. Research report: 2006 student census, Grades 7-12 – system overview. Toronto: Toronto Board of Education; 2007.
3. Statistics Canada. Census 2006. Long form. Ottawa; 2006.

Demographics, Service & Housing History Coding Legend

Q No.	SASCode	Answer Selection	SASVariableName
	[8888]	Subject skipped the questionnaire	
	[888]	Not applicable	
	[999]	No response to this question	
	[1]/[0]	Inclusive selection (checkbox): [1-Yes]/[0-No]	
1.		**What is your gender? Do you identify as:**	DSHHQ1
	[1]	Male	
	[2]	Female	
	[3]	Transgender	
	[4]	Transexual	
	[5]	Other	
	[98]	Declined	
2.		**Where were you born, that is, what country?**	DSHHQ2
	[1]	Albania	
	[2]	Afghanistan	
	[3]	Bangladesh	
	[4]	Canada	
	[5]	China	
	[6]	Guyana	
	[7]	Hong Kong	
	[8]	India	
	[9]	Iran	
	[10]	Jamaica	
	[11]	Pakistan	
	[12]	Philippines	
	[13]	Romania	
	[14]	Russia	
	[15]	Somalia	
	[16]	South Korea	
	[17]	Sri Lanka	
	[18]	Ukraine	
	[19]	United States	
	[20]	Vietnam	
	[21]	Yugoslavia	
	[22]	Other (specify)	
	[23]	Don't know	
	[98]	Declined	

2.a **If other (specify):**

(AlphaNumeric - Length: 60) DSHHQ2_a

2.b **Within Canada, what is your original home community, that is, where did you live after you were born and for your early years?**

(AlphaNumeric - Length: 255) DSHHQ2_b

2.c **When did you arrive in Canada?**

(AlphaNumeric - Length: 12) DSHHQ2_c

2.d **When you arrived in Canada, what was your status?** DSHHQ2_d

[5] Landed immigrant

[1] Visitor with intent to apply

[2] Student

[3] Temporary status (work permit, domestic help)

[4] Refugee Claimant (awaiting hearing)

[6] Other

[9] Don't know

[98] Declined

3. **Where were your parents born, that is, what countries?**

[1]/[0] Albania	DSHHQ3a
[1]/[0] Afghanistan	DSHHQ3b
[1]/[0] Bangladesh	DSHHQ3c
[1]/[0] Canada	DSHHQ3d
[1]/[0] China	DSHHQ3e
[1]/[0] Guyana	DSHHQ3f
[1]/[0] Hong Kong	DSHHQ3g
[1]/[0] India	DSHHQ3h
[1]/[0] Iran	DSHHQ3i
[1]/[0] Jamaica	DSHHQ3j
[1]/[0] Pakistan	DSHHQ3k
[1]/[0] Philippines	DSHHQ3l
[1]/[0] Romania	DSHHQ3m
[1]/[0] Russia	DSHHQ3n
[1]/[0] Somalia	DSHHQ3o
[1]/[0] South Korea	DSHHQ3p
[1]/[0] Sri Lanka	DSHHQ3q
[1]/[0] Ukraine	DSHHQ3r
[1]/[0] United States	DSHHQ3s
[1]/[0] Vietnam	DSHHQ3t
[1]/[0] Yugoslavia	DSHHQ3u

[1]/[0] Other DSHHQ3v

[1]/[0] Don't know DSHHQ3w

[1]/[0] Declined DSHHQ3x

3.a **Other specify:**

(AlphaNumeric - Length: 45) DSHHQ3_a

4. **What is the language you first learned to speak at home in childhood and still understand?** DSHHQ4

[1] Albanian

[2] Arabic

[3] Bengali

[4] Cantonese

[5] Mandarin

[6] Dari

[7] English

[8] French

[9] Greek

[10] Gujarati

[11] Hindi

[12] Korean

[13] Ojibway

[14] Persian (Farsi)

[15] Portuguese

[16] Punjabi

[17] Russian

[18] Serbian

[19] Somali

[20] Spanish

[21] Tagalog

[22] Tamil

[23] Urdu

[24] Vietnamese

[25] Other (specify)

[26] Don't know

[98] Declined

4.a **If other (specify):**

(AlphaNumeric - Length: 30) DSHHQ4_a

5. **What is your ethnic or cultural identity?** DSHHQ5

[1] Aboriginal (Skip to 5d)

[2] Asian - East* (e.g. China, Japan, Korea)

[3] Asian - South* (e.g. India, Pakistan, Sri Lanka)

[4] Asian - South East* (e.g. Malaysia, Philippines, Vietnam)

[5] Black - Africa* (e.g. Ghana, Kenya, Somalia)

[6] Black - Canada*

[7] Black - Caribbean Region* (e.g. Jamaica, Trinidad, Tobago)

[8] Latin American* (e.g. Argentina, Chile, Costa Rica)

[9] Indian-Caribbean* (i.e. Guyana with origins in India)

[10] Middle Eastern* (e.g. Egypt, Iran, Israel, Palestine)

[11] White - Canada

[12] White - Europe (e.g. England, Greece, Italy, Portugal, Serbia)

[13] Mixed Background - with at least 1 of groups marked with an asterisk(*) (specify)

[14] Mixed Background - without any of the groups marked with an asterisk(*) (specify)

[15] Other (specify)

5.a **If other (specify):**

(AlphaNumeric - Length: 60) DSHHQ5_a

5.b **If mixed (specify):**

(AlphaNumeric - Length: 60) DSHHQ5_b

5.c **If mixed (specify):**

(AlphaNumeric - Length: 60) DSHHQ5_c

5.d **If Aboriginal, are you (check all that apply):**

[1]/[0] Inuit DSHHQ5_da

[2]/[0] Metis DSHHQ5_db

[3]/[0] First Nations Status DSHHQ5_dc

[4]/[0] First Nations Non-status DSHHQ5_dd

[5]/[0] Indigenous from outside Canada DSHHQ5_de

[6]/[0] Other (specify) DSHHQ5_df

5.d1 **If other (specify):**

(AlphaNumeric - Length: 60) DSHHQ5_d1

6. **So just to confirm, how would you describe your ethnic or cultural background in your own words?**

(AlphaNumeric - Length: 300) DSHHQ6

7. **Do you identify yourself as Canadian? (You do not have to be born in Canada to think of yourself as Canadian).** DSHHQ7

[1] Yes

[0] No

[9] Don't know

[98] Declined

8. **Not counting kindergarten, how many years of school did you complete?**

(Numeric - Length: 2) DSHHQ8

9. **What is your level of education?** DSHHQ9

[1] Completed grade 4 or less

[2] Completed grade 5 to 8

[3] Attended High School, not completed

[4] Completed High School

[5] Attended business, trade, technical school (incl. CEGEP)

[7] Completed business, trade, technical school (incl. CEGEP)

[6] Attended University, not completed

[8] Completed University (Bachelor's degree)

[9] Attended Graduate School, not completed

[10] Completed Graduate School

[11] Don't know

[98] Declined

10. **Are you currently single... (read first 6 options):** DSHHQ10

[1] Single, never married

[2] Married

[3] Separated

[4] Divorced

[5] Cohabitating with a partner

[6] Widowed

[9] Don't know

[98] Declined

11. **How many children do you have under the age of 18 (19 in Vancouver)? (Include whether or not they live with you).** DSHHQ11

[0] 0

[1] 1

[2] 2

[3] 3

[4] 4

[5] >4

[9] Don't know

[98] Declined

11.a **How many of these children do you currently provide full or partial support to?**

(Numeric - Length: 2) DSHHQ11_a

11.b **What is your relationship to your child or children?**

[1]/[0] Biological parent DSHHQ11_ba
[1]/[0] Parent's partner (living together) DSHHQ11_bb
[1]/[0] Adoptive parent DSHHQ11_bc
[1]/[0] Foster parent DSHHQ11_bd
[1]/[0] Stepparent DSHHQ11_be
[1]/[0] Other adult relative (specify) DSHHQ11_bf
[1]/[0] Don't know DSHHQ11_bg
[1]/[0] Declined DSHHQ11_bh

11.c **If other adult relative (specify):**

(AlphaNumeric - Length: 60) DSHHQ11_c

12. **Have you worked continuously for at least one year in the past?** DSHHQ12

[1] Yes
[0] No
[9] Don't know
[98] Declined

13. **Have you ever had any wartime service in the military forces of Canada or its allies?** DSHHQ13

[1] Yes
[0] No
[9] Don't know
[98] Declined

14. **What is your current PRIMARY employment status?** DSHHQ14

[1] Unemployed
[2] Employed (including self-employed)
[3] Volunteer work, unpaid
[4] Employed in a special work program
[5] Retired
[6] Student
[7] Housewife/husband
[8] Other (specify)
[9] Don't know
[98] Declined

14.a **If other (specify):**

(AlphaNumeric - Length: 60) DSHHQ14_a

14.b **What is the main reason you are not working?** DSHHQ14_b

[1] Mental illness
[2] Physical illness
[3] Transportation problems
[4] Fear of loss of benefits
[5] Both mental and physical illness
[6] Other (specify)

14.c **If other (specify):**

(AlphaNumeric - Length: 60) DSHHQ14_c

14.d **Would you like to have a paid job in the community?** DSHHQ14_d

[1] Yes
[0] No
[9] Don't know
[98] Declined

14.e **What kind of job or jobs?**

(AlphaNumeric - Length: 90) DSHHQ14_e

14.f **How many hours per week?**

(Numeric - Length: 3) DSHHQ14_f

14.g **Wage per hour?**

(Numeric - Length: 6) DSHHQ14_g

14.h **Where are you studying?**

(AlphaNumeric - Length: 30) DSHHQ14_h

14.i **How many credits per semester are you currently registered for?**

(Numeric - Length: 3) DSHHQ14_i

14.j **How many hours per week are you currently registered for?**

(Numeric - Length: 2) DSHHQ14_j

15. **What are your current sources of income?**

[1]/[0] Earnings from regular work DSHHQ15a
[1]/[0] Longterm Disability (Private Insurer) DSHHQ15b
[1]/[0] Earnings from casual work DSHHQ15c
[1]/[0] Personal Needs Allowance (PNA (Ont.)) DSHHQ15d
[1]/[0] Unemployment insurance (EI or EIA in Man.) DSHHQ15e
[1]/[0] Selling papers, souvenirs, crafts DSHHQ15f
[1]/[0] Disability income (ODSP/CPPD (Ont.) IAPD (Man.)) DSHHQ15g
[1]/[0] Pan-handling DSHHQ15h

[1]/[0] Welfare/income assistance (OW (Ont.) w/PWD or PPMB status (BC)) DSHHQ15i

[1]/[0] Busking (entertaining for cash) DSHHQ15j

[1]/[0] Pension, incl. old age security, CPP, veteran's pension DSHHQ15k

[1]/[0] Squeegeeing DSHHQ15l

[1]/[0] Other (specify) DSHHQ15m

[1]/[0] Collecting/recycling (bottles, scrap metal etc.) DSHHQ15n

15.a **Other (specify):**

(AlphaNumeric - Length: 100) DSHHQ15_a

16. **In the study, we recognize that there may be other sources of income that you may have used to survive on the street. Please know that your confidentiality is protected if you would like to tell me about these sources of income, but like the other questions you do not have to tell me about them.**

(AlphaNumeric - Length: 200) DSHHQ16

17. **What was your total income, last month?**

(AlphaNumeric - Length: 10) DSHHQ17

18. **Do you have a provincial health card number?** DSHHQ18

[0] No

[1] Yes, in possession (write down,record in profile at end of interview, and destroy paper)

[2] Yes, but not here (ask 18.a)

[9] Don't know

[98] Declined

18.a **Is there a doctor''s office, hospital, shelter or housing office that you''ve been to recently that we would be able to get your health card number from?**

(AlphaNumeric - Length: 100) DSHHQ18_a

19. **In the past 5 years, have you been hospitalized for a mental illness at any time for longer than 6 months?** DSHHQ19

[1] Yes

[0] No

[9] Don't know

[98] Declined

20. **In the past 5 years, have you been hospitalized 2 or more times in any one year period for a mental illness?** DSHHQ20

[1] Yes

[0] No

[9] Don't know

[98] Declined

21. **Have you ever received treatment, counseling or harm reduction services for your use of alcohol or any drug, not counting cigarettes?** DSHHQ21

[1] Yes

[0] No

[9] Don't know

[98] Declined

22. **In the past 6 months, have you been arrested for criminal activity more than once, or been imprisoned at least once, or served probation or other community sanction?** DSHHQ22

[1] Yes

[0] No

[9] Don't know

[98] Declined

23. **In the past 6 months, did you spend one or more nights in a hospital, detox centre, jail or shelter?** DSHHQ23

[1] Yes

[0] No

[9] Don't know

[98] Declined

23.a **For the most recent stay, what is the name of the hospital, detox centre, jail or shelter?**

(AlphaNumeric - Length: 10) DSHHQ23_a

23.b **Approximately how many days were you in [location name]?**

(Numeric - Length: 3) DSHHQ23_b

23.c **For the stay before that, what is the name of the hospital, detox centre, jail or shelter?**

(AlphaNumeric - Length: 10) DSHHQ23_c

23.d **Approximately how many days were you in [location name]?**

(Numeric - Length: 3) DSHHQ23_d

23.e **And for the stay before that, what is the name of the hospital, detox centre, jail or shelter?**

(AlphaNumeric - Length: 10) DSHHQ23_e

23.f **Approximately how many days were you in [location name]?**

(Numeric - Length: 3) DSHHQ23_f

24. **When did you first become homeless (year)?**

(Numeric - Length: 4) DSHHQ24

25. **In your lifetime, what is the total amount of time you have been homeless (months)?**

(Numeric - Length: 3) DSHHQ25

26. **How long was your longest single period of homelessness (months)?**

(Numeric - Length: 3) DSHHQ26

27. **When did your last period of homelessness end?**

(AlphaNumeric - Length: 12) DSHHQ27
