# Supplementary material for: The association between experiencing homelessness in childhood or youth and adult housing stability in Housing First
Source: BMC Psychiatry. 2021 Mar 8;21:138. doi: 10.1186/s12888-021-03142-0 (PMC7938606; doi:10.1186/s12888-021-03142-0)
Supplement: Supplementary file 2 — Additional file 2:. Screener questionnaire; description of data: provides details of the questionnaire and lists its items. [file 12888_2021_3142_MOESM2_ESM.pdf]

The Association Between Experiencing Homelessness in Childhood or Youth and Adult Housing Stability in Housing First

Milad Parpouchi[a], Akm Moniruzzaman[a], Julian M. Somers[a]

[a] Somers Research Group, Faculty of Health Sciences, Simon Fraser University

Screener questionnaire

- Author: At Home/Chez Soi Study National Research Team
    - Author affiliation: Department of Psychiatry, University of Toronto
- The Mini International Neuropsychiatric Interview was also included in the questionnaire [1–4]
- Please see next page for questionnaire items

References

1. Sheehan D V, Lecrubier Y, Sheehan KH, Amorim P, Janavs J, Weiller E, et al. The Mini-International Neuropsychiatric Interview (M.I.N.I.): the development and validation of a structured diagnostic psychiatric interview for DSM-IV and ICD-10. J Clin Psychiatry. 1998;59 Suppl 20:22–33.
2. Amorim P, Lecrubier Y, Weiller E, Hergueta T, Sheehan D. DSM-III-R psychotic disorders: procedural validity of the Mini International Neuropsychiatric Interview (MINI). Concordance and causes for discordance with the CIDI. Eur Psychiatry. 1998;13:26–34.
3. Lecrubier Y, Sheehan D V., Weiller E, Amorim P, Bonora I, Sheehan KH, et al. The Mini International Neuropsychiatric Interview (MINI). A short diagnostic structured interview: reliability and validity according to the CIDI. Eur Psychiatry. 1997;12:224–31.
4. Sheehan D V., Lecrubier Y, Sheehan KH, Janavs J, Weiller E, Keskiner A, et al. The validity of the Mini International Neuropsychiatric Interview (MINI) according to the SCID-P and its reliability. Eur Psychiatry. 1997;12:232–41.

Screener Coding Legend

Q No.	SASCode	Answer Selection	SASVariableName
	[8888]	Subject skipped the questionnaire	
	[888]	Not applicable	
	[999]	No response to this question	
	[1]/[0]	Inclusive selection (checkbox): [1-Yes]/[0-No]	
1.	**Enter the Referring Source:**		
		(Numeric - Length: 4)	SCRNQ1
2.	**Ask them for their date of birth.**		
		(AlphaNumeric - Length: 12)	SCRNQ2
2.a	**To confirm, ask them if they are 18 years of age or older (19 in BC)?**		SCRNQ2_a
	[1]	Yes	
	[0]	No	
3.	**Are they currently Absolutely Homeless or Precariously Housed?**		SCRNQ3
	[1]	Yes	
	[0]	No	
4.	**Record Housing Status:**		SCRNQ4
	[1]	Absolutely Homeless	
	[2]	Precariously Housed	
5.	**Are they a client of ICM or ACT Program?**		SCRNQ5
	[1]	Yes	
	[0]	No	
6.	**Verbal expression of clearly false or bizarre ideas such as reporting references to self on TV or in newspaper or claiming to be someone he/she clearly is not.**		SCRNQ6
	[1]	Yes	
	[0]	No	
7.	**Appears to or reports hearing sounds of people talking when no one is around, or sees visions others do not see.**		SCRNQ7
	[1]	Yes	
	[0]	No	
8.	**Extremely difficult to understand because odd use of words, rambling, and/or sudden change of topics.**		SCRNQ8
	[1]	Yes	
	[0]	No	

9. **Indications of extreme sadness together with severe apathy or withdrawal.** SCRNQ9

[1] Yes

[0] No

10. **Engages in self harm activities such as slashing and/or frequent overdosing.** SCRNQ10

[1] Yes

[0] No

11. **Has written documentation of diagnosed non-substance-related mental disorder or written documentation of a psychiatric inpatient admission that meets the threshold for illness. Record diagnosis (es):** SCRNQ11

[1] Yes

[0] No

11.a **Current Major Depressive Episode** SCRNQ11_a

[1] Yes

[2] No

11.b **Current Manic Episode or Hypomanic Episode** SCRNQ11_b

[1] Yes

[2] No

11.c **Current PTSD** SCRNQ11_c

[1] Yes

[2] No

11.d **Current Panic Disorder** SCRNQ11_d

[1] Yes

[2] No

11.e **Current Mood Disorder with Psychotic Features** SCRNQ11_e

[1] Yes

[2] No

11.f **Current Psychotic Disorder** SCRNQ11_f

[1] Yes

[2] No

11.g **OTHER, specify:**

(AlphaNumeric - Length: 90) SCRNQ11_g

12. **Needs assistance to meet nutritional needs (e.g., unable to obtain/access meals even when opportunity is available).** SCRNQ12

[1] Yes

[0] No

13. **Needs assistance to maintain minimally adequate personal hygiene, (e.g., washing, grooming, oral hygiene).** SCRNQ13

[1] Yes

[0] No

14. **Needs assistance or is unwilling to access needed resources (e.g., financial benefits, clothes, appropriate accommodation or medical care).** SCRNQ14

[1] Yes

[0] No

15. **Needs assistance to acquire and maintain network of family, friends and peers (e.g., extremely isolated).** SCRNQ15

[1] Yes

[0] No

16. **Needs assistance to manage finances (e.g., unable to budget for basic daily living needs).** SCRNQ16

[1] Yes

[0] No

17. **Current Major Depressive Episode** SCRNQ17

[1] Yes

[0] No

18. **Current Manic Episode or Hypomanic Episode** SCRNQ18

[1] Yes

[0] No

19. **Current PTSD** SCRNQ19

[1] Yes

[0] No

20. **Current Panic Disorder** SCRNQ20

[1] Yes

[0] No

21. **Current Mood Disorder with Psychotic Features** SCRNQ21

[1] Yes

[0] No

22. **Current Psychotic Disorder** SCRNQ22

[1] Yes

[0] No

23. **Current Alcohol Dependence** SCRNQ23

[1] Yes

[0] No

24. **Current Substance Dependence** SCRNQ24

[1] Yes

[0] No

25. **Current Alcohol Abuse** SCRNQ25

[1] Yes

[0] No

26. **Current Substance Abuse** SCRNQ26

[1] Yes

[0] No

27. **Current Suicidality** SCRNQ27

[1] Low

[2] Moderate

[3] High

[4] No

28. **Were you in a special class in school?** SCRNQ28

[1] Yes

[0] No

[9] Don't know

29. **Did you ever get extra help with learning in school?** SCRNQ29

[1] Yes

[0] No

[9] Don't know

30. **Do you think you have or do you feel you have a learning problem or learning disability?** SCRNQ30

[1] Yes

[0] No

[9] Don't know

31. **Did anyone ever tell you that you have a learning problem or learning disability?** SCRNQ31

[1] Yes

[0] No

[9] Don't know
